# Galectin-7 Expression in the Placentas of Women with Gestational Diabetes Mellitus

**DOI:** 10.3390/ijms251810186

**Published:** 2024-09-23

**Authors:** Christina Teresa Seifert, Laura Unverdorben, Julia Knabl, Stefan Hutter, Simon Keckstein, Elisa Schmoeckel, Mirjana Kessler, Udo Jeschke, Sven Mahner, Thomas Kolben, Franziska Ganster

**Affiliations:** 1 Department of Obstetrics and Gynecology, University Hospital, LMU Munich, 81377 Munich, Germany; 2 Department of Pathology, University Hospital, LMU Munich, 81377 Munich, Germany; 3 Department of Obstetrics and Gynecology, University Hospital Augsburg, 86156 Augsburg, Germany

**Keywords:** galectin-7, gestational diabetes mellitus, pregnancy

## Abstract

Gestational diabetes mellitus (GDM) is a common condition during pregnancy. The prevalence of GDM is continuously increasing worldwide. Due to accessible diagnostic methods and a clear understanding of risk factors, GDM can be effectively diagnosed and managed. Galectins may influence immunomodulatory and inflammatory processes. This study examines the expression of galectin-7 in the placentas of women with gestational diabetes (GDM), compares it to its expression in healthy pregnancies, and evaluates the associated clinical outcomes. The placentas of 40 healthy women and 40 GDM placentas were included in the cohort. The expression level of galecin-7 was measured in the syncytiotrophoblast (SCT) and in the decidua of the placenta by immunohistochemistry and double immunofluorescence staining. The evaluation was performed by an immunoreactivity score (IRS). The study results show an increased expression of galectin-7 in the SCT and the decidua of GDM placentas as compared to the placentas of the control group. Elevated levels of galectin-7 were observed in both the nucleus and the cytoplasm. This study investigated the hypothesis that galectins are involved in pathophysiological processes of gestational diabetes. Statistical analysis of gene expression patterns confirmed that galectin-7 is indeed upregulated in GDM placentas. Further studies are needed to show the correlation of galectin-7 and the development and maintenance of gestational diabetes mellitus.

## 1. Introduction

Gestational diabetes mellitus is a growing global health issue. It is defined as a pancreatic inability to produce insulin, resulting in a glucose intolerance and high blood sugar levels during pregnancy. Maternal factors that predispose the development of GDM include a family history of type 2 diabetes mellitus, advanced maternal age, obesity, and ethnicity [1]. As the frequency of women suffering from diabetes and hyperglycemia during pregnancy is rising, the risks of developing severe pregnancy outcomes for the fetus are detected more often. These severe pregnancy outcomes are macrosomia, shoulder dystocia, pre-eclampsia, and hypertensive disorders [2]. It is estimated that the worldwide prevalence of GDM will increase to 4.4% by 2030 [3].

Furthermore, long-term consequences include a predisposition for metabolic syndrome, obesity, and the occurrence of diabetes later in life. 

Unfortunately, screening and treatment procedures vary across different regions of the world. This can cause underdiagnosis and undermanagement of the disease and emphasizes the need for a common approach [4].

Barodes et al. described galectins as proteins belonging to the lectin family, distinguished by their unique amino acid sequences and their affinity for β-galactoside sugars. Depending on their function, galectins appear in the nucleus, the cytoplasm, or the extracellular space. To date, more than 16 galectins have been identified in vertebrates and are classified based on their structure and location. The function of these proteins varies within the galectin family. In principle, galectins play an essential role in biological and immunomodulatory functions. These functions include cell–cell interactions, cell differentiation, inflammation, and metastatic processes [5,6]. Many studies have demonstrated the significant impact of galectins in various diseases, including autoimmune and inflammatory disorders. Interestingly, the inhibition of certain galectins can be therapeutically beneficial in infectious diseases by enhancing the immune response [7,8,9].

In obstetric diseases, previous studies could demonstrate that the expression of galectins changes. An example of this is the study by Chafetz et al., which revealed that the serum level of Placental Protein 13 (PP13), also known as galectin-13, is decreased during the first trimester in women later diagnosed with pre-eclampsia, intrauterine growth restriction (IUGR), and preterm deliveries. [10]. The decrease in galectin-13 could also be described in placental tissue in women suffering from GDM [11]. These studies suggest that galectins can influence obstetric disorders.

Compared to this, Jeschke et al. were able to show increased levels of galectin-1 and galectin-3 in pre-eclamptic and HELLP placentas. These results lead to the assumption that this upregulation may equalize the apoptotic reactions of maternal immune cells [12].

In analogy to the present study, the involvement of another member of the galectin group has already been analyzed in GDM placentas, which will be explained below.

It is suspected that galectin-4 plays a pro-inflammatory role in chronic intestinal diseases.

For this reason, Schrader et al. investigated the presence of galectin-4 in placental tissue and observed an elevated levels of galectin-4 in GDM placentas in comparison to healthy controls. This emphasizes the fact that galectins are involved in the development of chronic inflammation in GDM [13,14].

Due to its properties and the known influence of galectin-7 in various processes, this study focusses on galectin-7 in gestational diabetes.

Galectin-7 belongs to the human galectin group that contains a single carbohydrate-recognition domain (CRD) and forms homodimers. It was first described by Madsen et al. in 1995 as a keratinocyte-specific marker of the epidermis and other epithelia [15]. To date, the involvement of galectin-7 in molecular mechanisms is not exactly understood.

Sewgobind et al. provided a comprehensive overview of the influence of galectin-7 in apoptosis, cell migration, cell adhesion, re-epithelialization processes, and its role in human diseases, including cancer [16]. This emphasizes the versatility of galectin-7. In cancer diseases, the expression of galectin-7 varies depending on the type of cancer: elevated levels have been observed in esophageal squamous cell carcinomas and breast cancer cells, likely due to the ability of galectin-7 to protect cells from apoptosis [17,18]. Conversely, a downregulation of galectin-7 has been noted in prostate cancer cells [19].

Interestingly, galectin-7 is also recognized for its participation in both physiological and pathophysiological processes within the female reproductive system, including the menstrual cycle, pre-eclampsia, and miscarriages [16]. Evans et al. submit evidence suggesting that galectin-7 may function as an adhesion molecule in women, thereby facilitating trophoblast–endometrial epithelial cell adhesion. This hypothesis is supported by the observation of higher serum levels of galectin-7 in pregnant women with a history of miscarriages as compared to those with healthy pregnancies. Additionally, increased serum concentrations of galectin-7 have been observed in women who developed pre-eclampsia, likely as a consequence of oxidative stress [20].

Investigations of the expression of galectin-7 in placental tissue hypothesize that augmented galectin-7 acts during placental formation and causes placental inflammation and placental release of anti-angiogenic factors [21].

To date, the participation of galectin-7 in obstetric diseases is suggested, but the expression pattern of galectin-7 in GDM has not yet been analyzed.

In summary, current data regarding the variety of functions and involvements of galectins in inflammatory and metabolic processes qualifies galectin-7 as a candidate to investigate in gestational diabetes mellitus.

## 2. Results

### 2.1. Study Population

The study population consists of 80 expected women. At total of 40 healthy women were assigned into a control group, and 40 women diagnosed with GDM constituted the case group. For further analysis, clinical data were collected to specify differences within the groups.

Fetal gender was split equally into 20 male and 20 female newborns in each group.

Furthermore, maternal BMI data as a continuous variable were collected for both groups. Here, BMI is shown to be significantly elevated in the case group with 28.13 kg/m^2^ compared to 23.35 kg/m^2^ in the control group (*p* = 0.002). A significant difference was also shown within the fetal birthweights (*p* = 0.013). The mean birthweight in the case group was significantly higher (3611 g) as compared to the fetal birthweight in the control group, which was lower (3317 g).

Insulin therapy was necessary in 75% of women in the case group.

The differences in delivery mode were shown to be not significant, as the vaginal delivery mode was higher in both groups.

Table 1 summarizes these data.

To analyze galectin-7 expression in the placental tissue samples, the IRS was assessed in both the SCT, representing the fetal part of the placenta, and the decidua, representing the maternal part of the placenta.

Table 2 gives an overview of the IRS.

### 2.2. Galectin-7 Is Upregulated in the SCT of GDM Placentas

Our results indicate that galectin-7 is upregulated in the nuclear SCT of GDM placentas compared to healthy controls (*p* = 0.001), as well as in the cytoplasmic SCT of GDM placentas (*p* = 0.001). The *p*-values are statistically significant.

**Figure 1:**
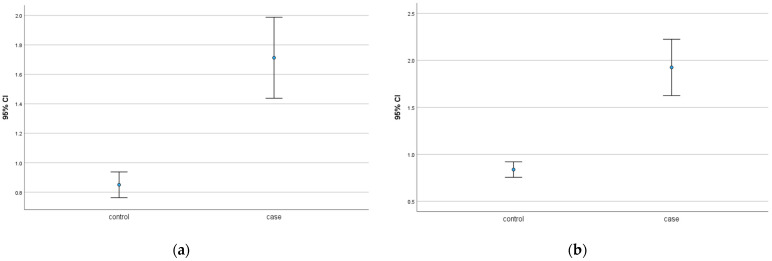
(**a**) Galectin-7 expression in the nuclear syncytiotrophoblast of healthy control versus GDM placentas. (**b**) Galectin-7 expression in the cytoplasmatic syncytiotrophoblast of healthy control versus GDM placentas.

The median IRS is 2.0 in the case group and 1.0 in the control group. Figure 1 underlines the results. 

### 2.3. Galectin-7 Is Upregulated in the Decidua of GDM Placentas

**Figure 2:**
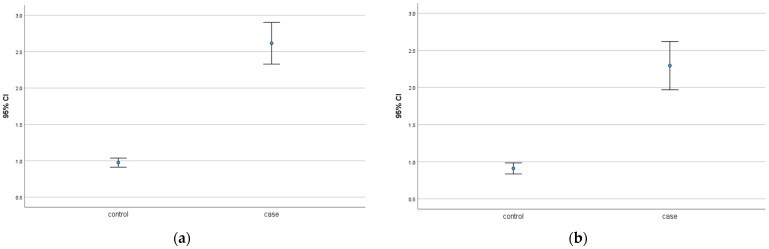
(**a**) Galectin-7 expression in the nuclear decidua of healthy control versus GDM placentas (**b**) Galectin-7 expression in the cytoplasmatic decidua of healthy control versus GDM placentas.

**Figure 3:**
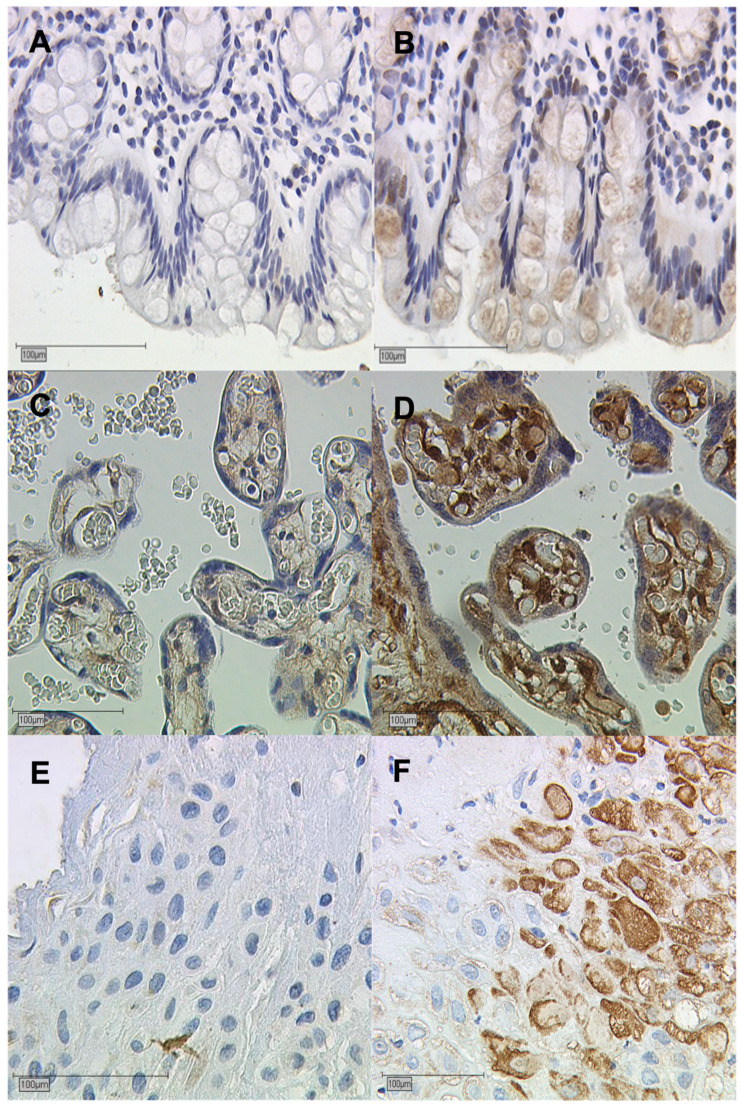
Immunohistochemical staining of galectin-7. (**A**) Negative control of human colon tissue; (**B**) Positive control of human colon tissue; (**C**) Galectin-7 staining in the syncytiotrophoblast of control placentas; (**D**) Galectin-7 staining in the syncytiotrophoblast of GDM placentas; (**E**) Galectin-7 staining in the decidua of control placentas; and (**F**) Galectin-7 staining in the decidua of GDM placentas.

This study investigated the expression of galectin-7 in the decidua of GDM placentas and healthy controls. Here, the findings suggest that there is a significant overexpression of galectin-7 in the nuclear decidua (*p* = 0.001) and the cytoplasmatic decidua of GDM placentas (*p* = 0.001). The median IRS is 3.0 in the nuclear decidua of GDM placentas and 1.0 in control placentas. For the cytoplasmatic decidua, the median was determined to be 2.0 in the case group and 1.0 in the control group. These results of galectin-7 are shown in Figure 2. Immunohistochemical staining images underline the findings in Figure 3. The scale bar equals 100 µm.

### 2.4. Visualization of Galectin-7 Overexpression in Immunofluorescence Double Staining

The double immunofluorescence staining technique was used to examine the localization pattern of galectin-7 overexpression in GDM placentas. Cytokeratin 7, a marker for extravillous trophoblast cells (EVT), was used in this study to differentiate between fetal and maternal cells. Microscopic analysis revealed co-expression of CK7 and galectin-7 in the same cell type. This confirms that the overexpression of galectin-7 likely occurs in the extravillous trophoblast.

**Figure 4:**
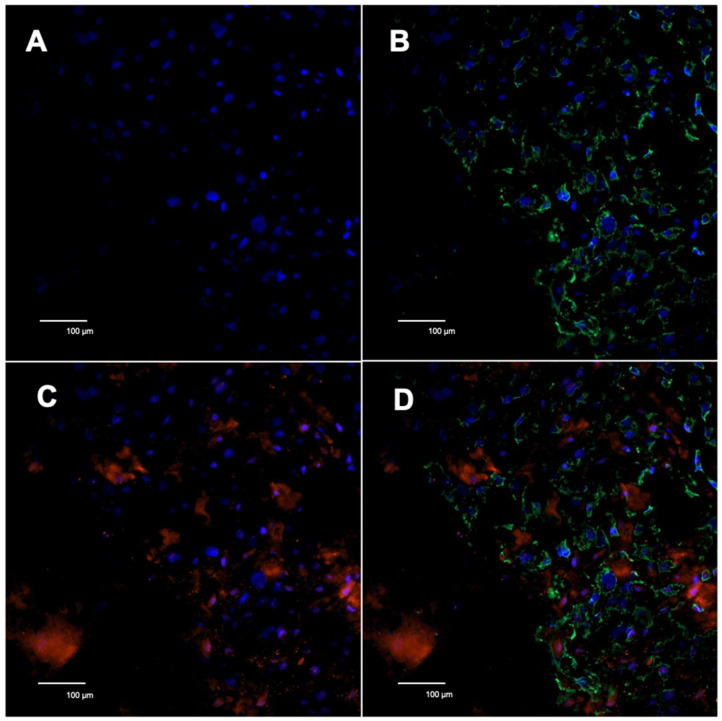
Double immunofluorescence phenotyping of decidual cells of the placenta in 20× magnification. Nuclei are stained with DAPI (blue). Galectin-7 is stained red. CK7 is stained green, marking the extravillous trophoblast (EVT). (**A**,**B**) Galectin-7 expression in control group placentas; (**C**,**D**) Galectin-7 expression in GDM placentas.

Figure 4 illustrates these results.

### 2.5. Multiple Regression Analysis

Fetal sex might be a risk factor for developing gestational diabetes. Therefore, a multiple linear regression analysis was conducted to eliminate potential interactions and confounding factors.

The linear regression model showed significance within the gender group for the expression of the galectin-7 in the nuclear SCT (*p* = 0.004) and the cytoplasmatic SCT (*p* = 0.005). On the contrary, the differences in the decidua were found to be insignificant. (*p* = 0.057).

Consequently, fetal gender could be seen as a confounder for placental galectin-7 expression in this study.

## 3. Discussion

Diabetes mellitus is caused by a dysregulation in carbohydrate metabolism; as defined by the American Diabetes Association, it results in hyperglycemia. In clinical practice, increased glucose or increased A1C (glycated hemoglobin) can be measured in the blood. The prevalence of GDM is increasing worldwide. It is one of the most common complications in pregnancy. The standard of care to screen for GDM is the 75 g OGTT between 24 and 28 weeks of gestation. The diagnosis is made when the following plasma glucose levels are reached: 92 mg/dL fasting glucose, 180 mg/dL after 1 h, and 153 mg/dL after 2 h. Pre-existing hyperglycemia is often only diagnosed during screening for GDM in pregnancy [22]. Therefore, it is recommended to identify diabetes preconceptionally in high-risk populations or before 24 weeks of gestation to reduce maternal and fetal risks in the peripartum period [22,23].

The pathogenesis of GDM is complex and the involvement and interactions of biomarkers remain only partially understood. Investigations into novel biomolecules have the potential to allow for the detection of women at risk of developing GDM and to deepen our understanding of the underlying pathogenic processes.

Ruszala et al. summarized a variety of molecules in the serum of women diagnosed with GDM in comparison to healthy controls. For example, GDF-15, a protein involved in inflammation and cell repair processes, is secreted in response to high blood glucose levels as a protective factor from glucose intolerance. Elevated concentrations were observed at the beginning of the third trimester in women with GDM.

Another molecule, the fatty acid binding protein, FABP4, is involved in lipid metabolism. It is illustrated that there is a correlation between a high serum level of FABP4 and an increased risk of GDM. Interestingly, inhibition of FABP4 can result in a relief of GDM symptoms by improving glucose and insulin sensitivity [24].

To date, the processes through which the interaction of these molecules with galectin-7 (examined in this study) influences the development of GDM remains unclear.

Since the group of galectins is known to be involved in various cellular functions, such as cell–cell interactions, cell-matrix adhesions, and immunomodulating and inflammatory processes, the aberrant expression of galectins in obstetric disorders is of great interest [9].

Previous studies could underline the variability of galectin expression in women suffering from miscarriages, HELLP syndrome, IUGR, GDM, or premature preterm rupture of membranes (pPROM). An overexpression of galectin-1 mRNA has been proven in the membranes of pPROM cases with histopathologically confirmed inflammation of the membranes. Similar results could be shown for galectin-3, which has a pro-inflammatory function by inhibiting the apoptosis of macrophages and monocytes [25,26].

Unverdorben et al. examined the expression levels of galectin-1, -2, -7, and -10 in placentas of miscarriages and figured out a decrease in these galectin members (except for galectin-3) in the placental tissue of abortions [27].

Additionally, it could be demonstrated that galectin-7 is expressed in the decidua, the endometrium, the EVT, and the SCT of the maternal–fetal interface, which leads to the assumption that a dysregulation can influence the development of pathological pregnancies [28].

Since dysregulation of galectins in GDM has been recently described, we identified galectin-7 as a potential molecule to clarify pathological processes in GDM [14,29,30].

The objective of this study is to evaluate the expression of galectin-7 in the placental tissue of women diagnosed with GDM compared to healthy controls. A differentiation of the expression levels in the nucleus and the cytoplasm of the placenta cells was performed. A significant increase in galectin-7 expression was observed in both the nucleus and cytoplasm of the SCT and decidua in GDM placentas through immunohistological staining and IRS evaluation. Double immunofluorescence visualization underlines the results. These data contribute additional insights into the potential mechanisms underlying the pathogenesis of GDM.

As previously mentioned, galectin-7 may participate in placental inflammation and the occurrence of pre-eclampsia by influencing the renin–angiotensin system [21,31].

This study confirms the dysregulation of another member of the galectin family in GDM, indicating that galectin-7 contributes to the inflammatory and metabolic effects associated with the development of GDM. As described above, the presence of galectin-7 in cancer types has been investigated. A correlation between the expression of galectin-7 in cancer and GDM and obstetric disorders has not yet been described, and further studies on this are recommended.

As already shown in the study of Ruszala et al., an examination of the serum level of galectin-7 is recommended when translating the findings into clinical practice to manifest the hypothesis [24]. To achieve this, further research is necessary to compare serum levels of galectin-7 with its placental expression and to investigate its potential as a marker for managing risk factors in pregnancies complicated by GDM.

Previous studies highlight the role of galectin-7 in hypertensive disorders, but clinical data from this study population lack information on co-existing pregnancy-related and non-pregnancy-related conditions, which could also contribute to the upregulation of galectin-7.

The reasons for a dysregulation of galectin-7 in GDM is still unknown.

However, this study provides a basis for further investigations to evaluate the co-regulation of galectin-7 and the variety of molecules that are known to be expressed in GDM cases to understand the complex functions in pregnancy-related diseases.

## 4. Materials and Methods

### 4.1. Study Design

For this study, ethical approval was obtained by the faculty of LMU Munich with Approval No. 337-06. All participants consented to this study project by giving informed consent.

The study population consists of 80 expected women. A total of 40 healthy women were assigned into a control group and 40 women diagnosed with gestational diabetes mellitus (GDM) between 2013 and 2015 were assigned to a case group. The diagnostic criteria to confirm GDM were based on the guidelines of the German Society of Diabetes Mellitus including the following parameters: The oral Glucose Tolerance Test (oGTT) was performed by all participants between pregnancy week 24 and 28. Identification of GDM was carried out by one increased measurement (fasting glucose >92 mg/dL, >180 mg/dL after one hour, and >153 mg/dL after two hours).

Additional data were collected in both groups. These included fetal gender, maternal body mass index (BMI), insulin therapy, birth mode, and fetal birthweight.

For the experimental study, part of placental tissue was removed directly after delivery from all placentas; the samples were a 2 × 2 × 2 cm tissue of a central cotyledon containing the following placental layers: maternal decidua, fetal syncytiotrophoblast, and amniotic epithelia. Placental tissue was fixed in a 4% buffered formalin solution and embedded in paraffin for subsequent analysis.

### 4.2. Immunohistochemical Staining

A detailed protocol for immunohistochemical staining was used [32]. After removing paraffin from placental tissue in a Roticlear bath (Carl Roth, Karlsruhe, Germany), a 3% H_2_O_2_ solution was applied to block peroxidase activity. Next, a high-pressure treatment with sodium citrate at pH 6.0 was performed for demasking the protein epitopes. For prevention of unspecific antigen–antibody interactions, a blocking solution (ZytoChem Plus HRP Polymer System, Zytomed Systems GmbH, Berlin, Germany) was applied. After this, incubation with a primary antibody (anti-galectin-7-antibody (polyclonal rabbit IgG, concentrate 0.05 mg/mL, NBP1-89690, Novus Biologicals, Minneapolis, MN, USA)) was performed, followed by dissolving in PBS at a 1:200 dilution for 16 h at 4 °C and treating with Post Block (Reagent 2, ZytoChem Plus HRP Polymer System mouse/rabbit, Zytomed) for 30 min. Finally, the application of Chromogen 3,3′-diaminobenzidine (DAB; Dako; Glostrup, Denmark) allowed for the visualization of galectin-7.

In the immunohistochemical approach, human colon tissue was selected for positive and negative control staining.

For evaluation, the semi-quantitative immunoreactivity score (IRS) was used to quantify staining intensity and the percentage of positively stained cells. Both parameters were multiplied to obtain the final IRS. The values were determined using an IRS ranging from 0 to 12 for each slide across four fields at 40× magnification. All samples were assessed under a Leitz Diaplan microscope with a minimum of 100 cells counted per sample. Two independent observers investigated the slides with this procedure.

### 4.3. Double Immunofluorescence

For double immunofluorescence staining, paraffin was removed, and a blocking solution was applied to the samples (Ultra V-Block, Thermo Scientific, Lab Vision, Fremont, CA, USA) for 15 min. This step is necessary to prevent any antigen–antibody interactions.

The next step included treatment with a primary antibody mixture with a secondary fluorescent antibody mixture for 30 min. Table 3 gives an overview of applied antibodies and complemented dilution and incubation information. Thereafter, slides were treated with mounting buffer (Vector Laboratories, Burlinghame, CA, USA), which contains DAPI to achieve nuclear counterstaining.

Analyzing and imaging were obtained with a fluorescent Axiokop photomicroscope and a digital Axiocam camera system (Zeiss, Oberkochen, Germany) under a 63-fold objective.

As Cytokeratin 7 (CK7) is a marker for extravillous trophoblast cells, performing double immunofluorescence of CK7 was used to differentiate between maternal and fetal cells.

### 4.4. Statistical Analysis

For statistical analysis, IBM SPSS Statistics (Version 26 for MAC, Armonk, NY, USA) was utilized. A Student’s *t*-test was performed for categorical data, and a Kruskal–Wallis test was used for data with continuous parameters. Multivariate analysis was conducted using a linear regression model. A *p*-value of <0.05 was considered to be significant.

## Figures and Tables

**Table 1:** Epidemiological and clinical data of the study population.

	GDM		Control		*p*-Value
**Gender**	*n*	%	*n*	%	
Male	20	50.0	20	50.0	
Female	20	50.0	20	50.0	
**Maternal BMI prior to pregnancy (kg/m^2^)**					
Underweight (<18.5)	0	0	4	10.0	0.116
Normal (18.5–24.9)	16	40.0	25	62.5	0.044
Overweight (25.0–29.9)	10	25.0	3	7.5	0.034
Obese (≥30.0)	12	30.0	5	12.5	0.056
**Insulin therapy**					
	30	75.0	0	0	
**Delivery mode**					
Vaginal	27	67.5	32	80.0	0.310
C-section	13	32.5	8	20.0	0.310
**Fetal birthweight (g)**					
Low birthweight (<3000)	1	2.5	2	5.0	0.556
Normal birthweight (3000–4000)	30	75.0	33	82.5	0.412
High birthweight (>4000)	9	22.5	5	12.5	0.239

**Table 2:** Immunoreactivity score, IRS.

		GDM			Control		*p*-Value
	Mean	Median	Modus	Mean	Median	Modus	
SCT nuclear	1.7125	2	1	0.85	1	1	0.001
SCT cytoplasmatic	1.925	2	1	0.8375	1	1	0.001
Decidual nuclear	2.6154	3	3	0.9744	1	1	0.001
Decidual cytoplasmatic	2.2949	2	4	0.9103	1	1	0.001

**Table 3:** Antibody features used for immunofluorescence.

Antibody	Dilution	Incubation	Manufacturer
Galectin-7—polyclonal Rabbit IgG	1:200	16 h at 4 °C	Novus Biologicals—NBP1-89690
CK7—Clone OVTL Mouse IgG	1:30	16 h at 4 °C	Novocastra—NCL-L-CK7-OVTL
CD31—Clone JC/70A Mouse IgG	1:50	16 h at 4 °C	Abcam—ab9498
Cy-2-labelled goat-anti-rabbit	1:100	30 min at RT	Dianova—115-226-062
Cy-3-labelled goat-anti-mouse	1:500	30 min at RT	Dianova—111-165-144

## Data Availability

Data is contained within the article.

## References

[1] Lende M., Rijhsinghani A. (2020). Gestational Diabetes: Overview with Emphasis on Medical Management. Int. J. Environ. Res. Public Health.

[2] Report of World Health Organization Consultation (2014). Diagnostic criteria and classification of hyperglycaemia first detected in pregnancy: A World Health Organization Guideline. Diabetes Res. Clin. Pract..

[3] Wild S., Roglic G. (2004). Global prevalence of diabetes: Estimates for the year 2000 and projections for 2030. Diabetes Care.

[4] Reece E.A., Leguizamón G. (2009). Gestational diabetes: The need for a common ground. Lancet.

[5] Barondes S.H., Cooper D.N. (1994). Galectins. Structure and function of a large family of animal lectins. J. Biol. Chem..

[6] Kasai K., Hirabayashi J. (1996). Galectins: A family of animal lectins that decipher glycocodes. J. Biochem..

[7] Arthur C.M., Dias Baruffi M. (2015). Evolving mechanistic insights into galectin functions. Methods Mol. Biol..

[8] Sehrawat S., Reddy P. (2010). Galectin-9/TIM-3 interaction regulates virus-specific primary and memory CD8 T cell response. PLoS Pathog..

[9] Liu F.T., Stowell S.R. (2023). The role of galectins in immunity and infection. Nat. Rev. Immunol..

[10] Chafetz I., Kuhnreich I. (2007). First-trimester placental protein 13 screening for preeclampsia and intrauterine growth restriction. Am. J. Obstet. Gynecol..

[11] Unverdorben L., Hüttenbrenner R. (2015). Galectin-13/PP-13 expression in term placentas of gestational diabetes mellitus pregnancies. Placenta.

[12] Jeschke U., Mayr D. (2007). Expression of galectin-1, -3 (gal-1, gal-3) and the Thomsen-Friedenreich (TF) antigen in normal, IUGR, preeclamptic and HELLP placentas. Placenta.

[13] Nio-Kobayashi J., Takahashi-Iwanaga H. (2009). Immunohistochemical localization of six galectin subtypes in the mouse digestive tract. J. Histochem. Cytochem..

[14] Schrader S., Unverdorben L. (2022). Overexpression of galectin-4 in placentas of women with gestational diabetes. J. Reprod. Immunol..

[15] Madsen P., Rasmussen H.H. (1995). Cloning, expression, and chromosome mapping of human galectin-7. J. Biol. Chem..

[16] Sewgobind N.V., Albers S. (2021). Functions and Inhibition of Galectin-7, an Emerging Target in Cellular Pathophysiology. Biomolecules.

[17] Zhu X., Ding M. (2010). Identification of galectin-7 as a potential biomarker for esophageal squamous cell carcinoma by proteomic analysis. BMC Cancer.

[18] Campion C.G., Labrie M. (2014). The CCAAT/enhancer-binding protein beta-2 isoform (CEBPbeta-2) upregulates galectin-7 expression in breast cancer cells. PLoS ONE.

[19] Labrie M., Vladoiu M. (2015). A mutation in the carbohydrate recognition domain drives a phenotypic switch in the role of galectin-7 in prostate cancer. PLoS ONE.

[20] Evans J., Yap J. (2014). Galectin-7 is important for normal uterine repair following menstruation. Mol. Hum. Reprod..

[21] Menkhorst E., Zhou W. (2020). Galectin-7 Impairs Placentation and Causes Preeclampsia Features in Mice. Hypertension.

[22] (2024). American Diabetes Association Professional Practice Committee, Diagnosis and Classification of Diabetes: Standards of Care in Diabetes—2024. Diabetes Care.

[23] Sweeting A., Wong J. (2022). A Clinical Update on Gestational Diabetes Mellitus. Endocr. Rev..

[24] Ruszala M., Niebrzydowska M. (2021). Novel Biomolecules in the Pathogenesis of Gestational Diabetes Mellitus. Int. J. Mol. Sci..

[25] Kolanowska D.G., Swietlicki A. (2021). The role of galectins in obstetrics with particular emphasis on premature preterm rupture of membranes. Ginekol. Pol..

[26] Than N.G., Kim S.S. (2008). Chorioamnionitis and increased galectin-1 expression in PPROM—An anti-inflammatory response in the fetal membranes?. Am. J. Reprod. Immunol..

[27] Unverdorben L., Haufe T. (2016). Prototype and Chimera-Type Galectins in Placentas with Spontaneous and Recurrent Miscarriages. Int. J. Mol. Sci..

[28] Chen M., Shi J.L. (2022). Galectins: Important Regulators in Normal and Pathologic Pregnancies. Int. J. Mol. Sci..

[29] Buschmann C., Unverdorben L. (2023). Galectin-10 Expression in Placentas of Women with Gestational Diabetes. Curr. Issues Mol. Biol..

[30] Buschmann C., Unverdorben L. (2024). Placental expression of inflammatory Galectin-12 is associated with gestational diabetes. J. Reprod. Immunol..

[31] Menkhorst E., Zhou W. (2022). Galectin-7 dysregulates renin-angiotensin-aldosterone and NADPH oxide synthase pathways in preeclampsia. Pregnancy Hypertens..

[32] Hutter S., Knabl J. (2015). Fetal gender specific expression of tandem-repeat galectins in placental tissue from normally progressed human pregnancies and intrauterine growth restriction (IUGR). Placenta.

